# Bicycle use in Latin American cities: changes over time by socio-economic position

**DOI:** 10.3389/frsc.2023.1055351

**Published:** 2023-04-17

**Authors:** Ione Avila-Palencia, Olga L. Sarmiento, Nelson Gouveia, Alejandra Jáuregui, Maria A. Mascolli, Anne D. Slovic, Daniel A. Rodríguez

**Affiliations:** 1 Centre for Public Health, School of Medicine, Dentistry and Biomedical Sciences, Queen’s University Belfast, Belfast, Northern Ireland, United Kingdom; 2 Urban Health Collaborative, Dornsife School of Public Health, Drexel University, Philadelphia, PA, United States; 3 School of Medicine, Universidad de los Andes, Bogota, Colombia; 4 Department of Preventive Medicine, University of São Paulo Medical School, São Paulo, Brazil; 5 Department of Physical Activity and Healthy Lifestyles, Center for Health and Nutrition Research, Instituto Nacional de Salud Pública (INSP), Cuernavaca, Mexico; 6 School of Public Health, University of São Paulo, São Paulo, Brazil; 7 Department of City and Regional Planning and Institute for Transportation Studies, University of California, Berkeley, Berkeley, CA, United States

**Keywords:** bicycle, Latin America, cities, education, income, socio-economic (personal) factors, survey

## Abstract

**Introduction:**

We aimed to examine utilitarian bicycle use among adults from 18 large Latin American cities and its association with socio-economic position (education and income) between 2008 and 2018.

**Methods:**

Data came from yearly cross-sectional surveys collected by the Development Bank of Latin America (CAF). A total of 77,765 survey respondents with complete data were used to estimate multilevel logistic regression models with city as random intercept and year as random slope.

**Results:**

Individuals with high education and high-income levels had lower odds of using a bicycle compared with participants with lower education and income levels. These associations, however, changed over time with the odds of bicycle use increasing for all groups, especially among individuals with the highest education and income levels.

**Discussion:**

Our results confirm the broadening appeal of bicycling across socioeconomic positions in several Latin American cities and reinforce the importance of considering policies aimed at supporting and enhancing bicycle travel for all users.

## 1 Introduction

Bicycle use for transport has been associated with health, environmental, and societal benefits such as lower cardiovascular risk, lower body mass index, better physical and mental wellbeing, improving social interaction, and helping reduce air pollution, greenhouse gas emissions, and noise (de Nazelle et al., 2011; Oja et al., 2011; Brand et al., 2013; Martin et al., 2014; Mytton et al., 2016; Celis-Morales et al., 2017; Avila-Palencia et al., 2018; Dons et al., 2018). As a result, nowadays planners and policy-makers are promoting bicycle use as a healthy and sustainable mode of transportation and recreation in cities (Gatersleben and Appleton, 2007; World Health Organization Regional Office for Europe, 2022).

Physical environment factors such as distance, parking, and safety determine the initial feasibility of bicycling, with behavioral expectations, socio-cultural norms and beliefs (Heinen et al., 2010), and overall appeal (e.g., bicycling being recognized as sustainable and healthy) contributing to making it more or less attractive (Götschi et al., 2017).

At the individual level, personal and household demographic are likely to modify the importance of infrastructural and social interventions to increase bicycling (Piatkowski and Bopp, 2021), with indicators for individual-level socio-economic position like education, occupation and household income emerging as important ones (Rachele et al., 2015). However, behavioral expectations, and cultural norms around mobility differ across cultures and cities (Heinen et al., 2010) and are likely to change with time. Therefore, associations between individual factors such as socio-economic position and bicycling are also expected to change over time together with infrastructural policies (Parra et al., 2018; Iglesias et al., 2019).

Bicycles have had a changing social status in different parts of the world and in different historical periods. In the late 19th century, mainly in Europe, North America and other Anglophone parts of the world (e.g., Australia and New Zealand), middle and upper-class residents dominated bicycling, associating it with modernity and technological progress (Oldenziel and Albert de la Bruhèze, 2011; Longhurst, 2015). In the US, among certain groups bicycle use was associated with a movement toward increased gender equality (Maclaran and Kravets, 2018). With time, bicycles became safer and started to appeal to a broad set of users, ages, and abilities (Oldenziel and Albert de la Bruhèze, 2011; Longhurst, 2015).

In the early 20th century, manufacturing made bicycles less expensive and more accessible. Bicycles remained common but became less fashionable to elites, who moved on to more “modern” things: the automobile (Norcliffe, 2001). During and after World War II, the popularity of bicycles grew in response to gas rationing, war damages, scarcity, and poverty. The war-related experience reinforced the posterior image of the bicycle as a poor person’s mode of transport and often were a reason for the refusal to ride a bicycle. By contrast, the automobile was associated with affluence and freedom (Longhurst, 2015). This was reinforced by the postwar abundance of inexpensive fuel in many western economies as well as infrastructure investments that made automobile use more convenient and beneficial (Oldenziel and Albert de la Bruhèze, 2011). The success of the motor vehicle industry in occupying and dominating the public’s imagination as well as the increasing wealth and purchasing power of the middle classes, led to the dominance of the automobile. Only the very young, the strong, and those without enough resources to own an automobile, would ride bicycles.

After the oil embargo of the early 1970s, bicycle lanes were adopted as symbols of sustainability in some Western European cities, beginning a new period of bicycling promotion (Oldenziel and Albert de la Bruhèze, 2011). A few European countries promoted bicycling heavily with dedicated infrastructures (e.g., The Netherlands, Denmark), while other countries followed slowly, often decades later.

In parallel with these changes in bicycling, the sociodemographics of bicycling also diverges across cities and countries. Nowadays, bicyclists in European cities tend to have higher education and higher employment rates than non-cyclists (Raser et al., 2018). By contrast, in the US those earning <$35,000 per year and living in dense residential areas are more than 10 times as likely to travel by bicycle than others (The League of American Bicyclists, 2013). Yet, when the option to drive is available and affordable, low-income individuals, and immigrants in particular, are less willing to use a bicycle for any purpose. It has been suggested that, among those with private vehicles, high income households are more likely to bicycle in comparison to those from low-income households (Dill et al., 2006; Parkin et al., 2007).

Activities to encourage bicycling have been adopted and replicated in Latin America. For example, Bogota (Colombia) experienced an increase in bicycle share of trips from 0.58% in 1996 to 9.10% in 2017 (Rosas-Satizábal and Rodriguez-Valencia, 2019). The success of Bogota’s promotion of bicycling has been the result of several milestones. First, was the creation of the Ciclovia (open streets) in 1974 (a weekly car-free program which opened space for walking, bicycling, skating, and jogging in the city on working days). Second was the start of a decade of significant bicycle promotion activities through the mayor’s City Plan. And third, between 2012 and 2019, when there was a large investment to build 145 kilometers of bicycle lanes throughout the city (Rosas-Satizábal and Rodriguez-Valencia, 2019).

Despite these promotional efforts, it appears as if bicycle use is still traditionally associated with lower socio-economic position in Latin America. There are limited studies in Latin America and the Caribbean, with the available evidence concentrated in just a few countries (de Sá et al., 2017). This despite the fact that many cities have invested in bicycle infrastructure and support bicycle-friendly policies in the last decade (Banco Interamericano de Desarrollo, 2015). A study in Bogota found that most bicycle paths users reported living in areas of lower socio-economic position, had lower educational attainment, and did not own cars (Torres et al., 2013). Similar results have been found in country-level analyses for Colombia and Chile, where adults with lower socio-economic position showed the highest prevalence of bicycle use for transport (González et al., 2014; Aguilar-Farias et al., 2019). Although there is increasing awareness among decision-makers regarding the importance of bicycles for sustainable transport, this awareness has not permeated to lay citizens. Mirroring trends in other countries, this view seems to be changing as population subgroups have begun adopting an image of bicycling as trendy and a-la-mode (Pardo et al., 2021) despite recurrent concerns about safety. The degree to which such perceived changes in bicycle use accurately reflect overall changes in behaviors across subgroups remains to be examined. This is important because the motivations for cycling, and hence the policies to encourage it are likely to differ across subgroups.

In this study, we aimed to evaluate how socio-economic position, measured using individual education and income level, is associated with bicycle use among adults in Latin American cities and if those associations vary across a 10-year period. We hypothesized that individuals with higher socio-economic position are less likely than individuals with lower position to use a bicycle to access their main activities, but that the probability of using a bicycle has been increasing over time more rapidly for individuals from higher socioeconomic position compared to individuals from lower socioeconomic position.

## 2 Materials and methods

### 2.1 Study design and population

This is a study of repeated cross-sections of adults who participated in yearly surveys conducted by the Development Bank of Latin America (CAF, for its acronym in Spanish of Corporación Andina de Fomento). The surveys span a decade, from 2008 to 2018, and included 18 cities in Latin America though not all cities are present every year (Table 1). In terms of the design, for each city and year a representative sample of survey respondents was identified using a stratified clustered probabilistic sampling approach, with a cluster representing a neighborhood block. Five individuals, one per dwelling, were randomly selected for each block. One individual was interviewed per dwelling. The sampling was designed for an error margin of 5% with a 95% level of confidence. Additional details about the sampling design can be found elsewhere (Banco de Desarrollo de America Latina, 2014, 2018).

The survey collects demographic and socio-economic information from respondents and a set of household level characteristics. From 2008 to 2014 eligible respondents were adults 25–65 years old; in 2015 eligible respondents were adults 15–55 years old; and from 2016 to 2018 eligible respondents were adults 20–60 years old. To have comparable age categories for all years we used participants 25–55 years old. The survey data is open access and can be found at scioteca.caf.com. The survey contains yearly modules measuring access, quality, spending and satisfaction in urban transport services, security, garbage collection, water and sanitation, electricity, and housing. Additionally, each year special survey modules are incorporated according to the topic addressed in CAF’s Economy and Development Report.

### 2.2 Bicycle transport use

The CAF surveys assessed the use of transport modes, including bicycles by asking respondents to select “which transport modes do you generally use to get to your main destination in a regular day (work/study/routine destination).” Bicycles were among the response options available, and multiple responses were allowed to account for multimodal trips. The question wording and response options changed slightly from year to year, but responses always included the option “bicycle.” For this study we used responses to the bicycle option, resulting in a dichotomous variable (yes/no) for each respondent in each survey year. In 2015 the sample of Montevideo (Uruguay) presented complete missing data for the transport question and thus the city was excluded for that year.

### 2.3 Socio-economic position

We measured socio-economic position using individuals’ education and income level as the main predictors in the study. Education level was assessed by asking: “What is the highest educational level attained by you?” The answers had multiple options, which changed slightly depending on the year of the survey. For this study we created three categories harmonizing all response options from the different years, resulting in: (1) Less than high school, (2) High school or professional level, (3) College level or higher.

Individual income was measured with the question: “What is your normal monthly income?” However, the question changed slightly depending on the year of the survey. For example, surveys for 2010–2014 asked “What is your normal monthly income for all the jobs you do?; ” while surveys for 2015 to 2018 asked “What is your normal monthly income for the main job you do?” Respondents gave the amount using the local currency. If the participant did not give information spontaneously, the interviewer read different amounts in US dollars converted to local currency using the average official exchange rate from the month prior to the application of the questionnaire. Given differences in cost of living and inflation across years, income variables were adjusted for difference in purchasing power using the purchasing power parity conversion provided by the International Monetary Fund’s World Economic Outlook Database for each survey year.

Consistent with our desire to develop indicators of socioeconomic position, we created a variable of personal income based on the distribution within each year so that each category reflected as close as possible an income quartile for that year (low, medium- low, medium-high, high). In that way the different levels have different values each year depending on the distribution observed that year, but the categories still capture the relative position of each respondent. For the surveys from 2008 to 2012 and 2014 the values were: low equivalent to ≤$200, medium-low equivalent to $201–$400, medium-high equivalent to $401–$800, and high equivalent to ≥$801. For the survey from 2013 the values were: low equivalent to ≤$100, medium-low equivalent to $101–$400, medium-high equivalent to $401–$800, and high equivalent to ≥$801. And for the surveys from 2015 to 2018 the values were: low equivalent to ≤$400, medium-low equivalent to $401–$800, medium-high equivalent to $801–$1,600, and high equivalent to ≥$1,601. Since more than 40% of observations were missing income data, we did not use multiple imputation. Therefore, analyses of income are restricted to 60% of the sample.

### 2.4 Covariates

We included a set of variables that may be confounders of our main associations of interest and were deemed relevant with a directed acyclic graph we developed (Supplementary Figure A). Age was self-reported and gender was identified by the interviewer by observation. As sensitivity analyses, we also included kilometers of bicycle infrastructure (separated bicycle lanes) as they can be an important predictor of bicycle use (Mueller et al., 2018). Data for kilometers of bicycle infrastructure in the city over time were collected from multiple sources (Supplementary Table A) and ranged from 2008 or 2013 until 2018. We used linear interpolation to estimate yearly bicycle lane kilometers between the two time points for each city assuming a constant rate of growth in the intervening years. For cities that had 2013 as starting point, values were extrapolated linearly to 2008 in a similar way.

### 2.5 Statistical analyses

Multilevel mixed effects logistic regression models with city as random intercept and year as fixed effect and random slope were used to estimate the associations of education level and income with bicycle use. The equation can be found in Supplementary Table B. We included year as fixed effect because we wanted to capture the general trend of calendar year and since we accounted for city specific time effect, we also included year as random slope. The different associations were assessed running single predictor and multiple predictor models. Three models were fitted: (0) unadjusted models with the respective predictor and the outcome; (1) models adjusted by age and gender; and (2) models adjusted by age, gender, and bike lane kilometers (sensitivity analysis detailed in the Supplementary material).

To evaluate whether associations between socio-economic position and bicycling varied across a 10-year period we estimated models 1 and 2 for each socio-economic predictor (education and income) and created an interaction between the predictor and year. To illustrate our results, we estimated the marginal predicted probabilities at mean values for age and bike lanes kilometers, and woman for gender. Due to the higher percentage of bicycle use in 2015 compared to the other years, we run sensitivity analyses without 2015 data.

All models were estimated with a complete case analysis. In all contrasts a significance value of p < 0.05 was considered. All analyses were conducted in Stata version MP 15.1 (StataCorp LP, Texas USA).

## 3 Results

Table 2 shows the characteristics of the study participants and kilometers of bicycling infrastructure by survey year. The sample sizes range from 5,321 participants (in 2009) to 8,558 participants (in 2011). The mean age of the respondents is around 38 years and the gender distribution across the years is even. The most frequent educational attainment is having completed high school and/or having a professional/technical degree. The least frequent education level in all surveys is college or higher degree. Individual income has a high percentage of missing values (>37%) in all survey years. The distribution of the sample between the individual income categories fluctuates slightly, but in general the predominant categories are medium-low and medium-high. There is an overall increase in bicycle infrastructure from 2009 to 2018. The reason for the drop in the mean number of km of bicycling infrastructure in 2010 is because in that year Panama City was included in the sample, increasing considerably the sample size (adding 530 participants) and being a city with a low number of km of bicycling infrastructure (4.28 km). Most respondents do not use a bicycle as a travel mode to get to their main travel destination in a routine day, from 86.60% in 2015 to 98.50% in 2011. Across cities we can see low percentages of bicycle use (1.50%–13.40%), being Buenos Aires, Cordoba, Bogota, and Montevideo cities with higher percentages (Supplementary Table C). We observe an important increase in bicycle use in 2015, which we attribute to the data from that year from Montevideo and the fact that the bicycle use question was presented as an only one answer question, while the other years the survey presented the transport question as a multiple answer, this could have distorted the results. Aside from this observation and the fact that the percentages of bicycle use are low, overall, bicycling is increasing over time.

Table 3 shows associations of education and individual income level with bicycle use. Most of the analyses showed statistically significant inverse associations between high socio-economic position and bicycle use. Participants with an education level of “College or higher” or “High school or professional degree” had lower odds of using a bicycle compared with participants with an education level “Lower than high school” in models that adjusted for age and gender. Regarding income levels, we found that participants with “medium-high” and “high” income levels had lower odds of using a bicycle compared with participants with “low” income. Individuals with “medium-low” income levels also had lower odds of bicycling than “low” income but the coefficient was not statistically significant. Supplementary Table D in the appendix shows the results of models including income and education predictors simultaneously. “College or higher” education remained significantly inversely associated with bicycle use only in the model without covariates and income levels presented similar results as the single predictor models, being participants with “medium-high” and “high” income those with lower odds of using a bicycle compared with participants with “low” income. We found the same result after adjusting by bicycle infrastructure (Supplementary Table E) and considering all the different sample sizes (Supplementary Tables F–K).

**Figure 1:**
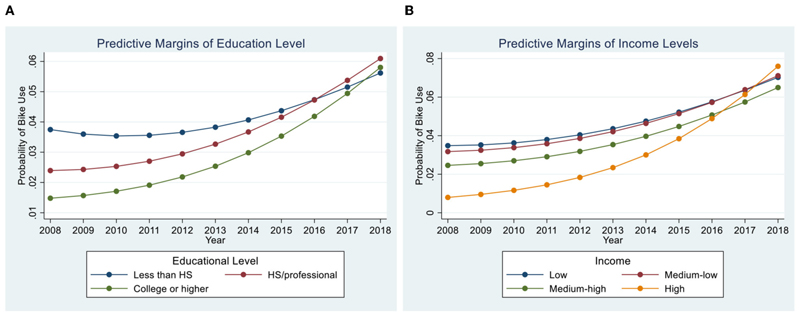
Predictive marginal probabilities of bike use by education and income level over time in CAF surveys of select Latin America cities. Models after controlling for age and gender (model 1). HS, high school. **(A)** Education level single predictor model after controlling for age and gender (model 1). Global test interaction *p*-value < 0.001. 95% CIs are not displayed for clarity. **(B)** Income level single predictor model after controlling for age and gender (model 1). Global test interaction *p*-value < 0.001. 95% CIs are not displayed for clarity.

Figure 1 shows how the predicted probability of using a bicycle changes by education and income level over time. In all figures there is an increase in the probability of bicycle use over time across all education and income groups. In Figure 1A the probability of bicycle use is higher over time across all education levels, but the increase is especially pronounced among individuals in the highest educational levels. In 2018, individuals with “high school or professional” and “college or higher” educational attainment have higher probability of bicycle use compared to those with education “lower than high school.” Figure 1B shows an increase in the probability of bicycle use over time, also most pronounced for individuals with high incomes. Figures based on multiple predictors models showed the same trend (Supplementary Figure B). We found the same trend after adjusting by bicycle infrastructure (Supplementary Figure C) and after using identical samples across all models (Supplementary Figures D–F).

## 4 Discussion

We examined associations between bicycle use and individual socio-economic position (education and income) while adjusting for relevant confounders in a sample of adults in 18 Latin American cities through up to 10 years of cross-sectional surveys. We found a statistically significant inverse associations between bicycle use and socio-economic position indicators. We also found that bicycle use is increasing over time across all groups, especially in individuals with higher socio-economic position.

However, the association between income and bicycle use was less consistent. Some authors have found a positive association between income and bicycle use whereas others do not find an association (Acharjee and Sarkar, 2021).

Our results though are in agreement with results of previous studies done in US and Latin American cities. In studies carried in the US, Colombia and Brazil having low income, poverty, having lower educational attainment, and not owning a car have been associated with cycling (The League of American Bicyclists, 2013; Torres et al., 2013; Lee et al., 2018; Torres-Freire et al., 2018). Bicycling continues to be a relatively popular low-cost mobility travel alternative for low income residents. Yet, we also found a considerable increase in the probability of cycling for high income individuals in the cities studied. The latter is more consistent with studies in European cities showing an association between high education and higher levels of cycling. In a study with a sample from Flanders (Belgium), higher education was associated with more cycling to work (De Geus et al., 2008). Also, in a multi-city European project, the share of cyclists with higher education and being employed was higher compared to non-cyclists (Raser et al., 2018).

The overall increase in bicycling observed in the Latin American cities observed may be partially explained by individual, environmental, and social factors. One possible explanation could be that with increasing congestion in Latin American cities (Wang et al., 2021), individuals may be considering other travel options to reach destinations, like bicycling. Similarly, concerns about personal and environmental health may be a heightened motivation for bicycling. In a study with a highly educated sample, those who already bicycled valued health-related benefits, its contribution to lower environmental degradation, and its role as a means for physical activity participation (Sener et al., 2009). These results are in line with the suggestion that those who perceive bicycling as a form of increasing their physical activity levels are more likely to ride a bicycle (Akar and Clifton, 2009).

The built environment, and especially changes in the presence and quality of bicycling infrastructure, may also have contributed to the observed increases in bicycling. Prior research has shown positive associations between bicycling network length and percentage of bicycling trips (Habib et al., 2014; Schoner and Levinson, 2014; Marqués et al., 2015; Schoner et al., 2015; Buehler and Dill, 2016) and with bicycling infrastructure being a crucial factor for preferring the bicycle as a transport mode (De Geus et al., 2008; Heesch et al., 2015; Mertens et al., 2016a,b; Torres-Freire et al., 2018). Thus, it is likely that the general increase in bicycling is partly the result of increases in bicycling infrastructure in the study cities. Beyond infrastructure, there may be many other policies, promotional activities, and programming that could contribute to the observed increases. For example, political leadership, a bicycle culture, effective advocacy, and clear city-planning documents where bicycle commuting is a goal, are other noteworthy explanations (Rosas-Satizábal and Rodriguez- Valencia, 2019). Indeed, the heterogeneity in bicycle use across the 18 cities studies (Supplementary Table C) suggest that city-specific factors are likely contributors to explain bicycle use.

Social norms, attitudes, and values can also be important factors that may explain differences across cities and among groups within cities. More bicycling may result from positive perceptions of bicycling. If an individual’s social surroundings have a positive opinion of bicycling, there is a higher chance that the individual will also have a positive opinion (Heinen et al., 2010). Accordingly, it has been suggested that social comparison, social image, and prestige are important social factors explaining individual’s intention to bicycle (Cepeda Zorrilla et al., 2019). Thus, the changes over time observed for different income groups can also reflect a cultural change toward the bicycle use in Latin American cities.

Increasing bicycling by more privileged individuals reinforces concerns about neighborhood change, gentrification, and displacement. In some places, bicycling and its associated infrastructure have become material symbols of neighborhood change, foregrounding social inequalities, and catalyzing neighborhood voices and discomfort from local residents. This has sometimes resulted in opposition to bicycle lane infrastructure, leading to discussions about culturally and locally appropriate bicycle interventions (Hoffmann, 2016; Lubitow et al., 2016). In these contexts, bicycling can be seen as a global force that responds to the logic of economic development by encouraging gentrification, increased property values, displacement, and redevelopment–a view readily adopted by local urban elites, planners, and decision-makers (Hoffmann and Lugo, 2014). The significant increases in bicycle use among individuals with high incomes and high educational attainment suggests that planners should pay close attention to how bicycling is presented and represented as it increases in popularity, and how plans to support bicycle draw from voices often ignored in planning processes. When investing in transport improvements including cycling infrastructure, especially in places like Latin America, planners should anticipate and address possible unintended outcomes and widening social inequalities that may result.

Bicycling continues to be an important mode of transportation for individuals in the lowest socio-economic position in Latin American cities. Even though the steepest increase in bicycling was by individuals of highest socio-economic position, bicycling across all groups increased. Lower income and lower education residents may have fewer mobility options and may be more sensitive to public transportation expenses. As documented elsewhere, for them bicycling provides significant mobility, physical activity, and financial benefits. Many users may be using the bicycle not as a choice, but as a necessity Depending on their location and design, infrastructure investments and programming are likely to benefit these users.

This study had several strengths. First, to our knowledge, this is the most extensive study in terms of sample size and duration examining associations between the socio-economic position and bicycle use over time. Second, we explored these associations using data from participants from many large and diverse cities from Latin America, with different socio-cultural characteristics and travel behaviors. Third, having access to data for repeated years from many cities allowed us to consider changes over time, and address critical questions about trends in use for different groups. Fourth, our measure of bicycle use is not limited to bicycle use as the main mode of travel for a given trip, or only as a feeder or distributor to other modes such as bus or train systems. We measure bicycling more comprehensively, considering all possible uses within a trip. Other reports about Latin American cities suggest a bicycle mode share that is always <10 percent (Ríos et al., 2015), but they focus narrowly on bicycles as the main mode of transportation to reach destinations. Finally, although we had significant missing data for individual income, income and education are highly positively associated (Supplementary Tables I,J). Therefore, education could be considered a proxy for income; we also observe that the results for education and income have similar trends over time.

Our study had some limitations too. First, our study used repeated cross-sectional surveys. This design, therefore, is not well suited to address causality. Second, all the measurements were self-reported using questionnaire data leading to possible measurement and misclassification error. Third, travel behaviors are complex, determined by many factors at many levels. To examine effects of single factors in more detail, we would have to observe the behavior in more detail and consider more potential determinants of those behaviors. Fourth, there may be other important factors such as city-specific policies around auto parking, public transportation fares, or vehicle circulation restrictions that may interact with socio-demographic characteristics in explaining whether individuals use a bicycle for utilitarian trips and that might explain observed trends. Our bicycle use data for 2015 seemed unusual, but our results held even after excluding that year from our analyses (Supplementary Table F). Fifth, our measure of bicycle infrastructure did not consider its quality and the linear interpolation for missing years may have introduced additional error. Sensitivity analyses (Supplementary Tables H, I; Figures E, F) showed that our results were robust to excluding observations with missing infrastructure data. Finally, the individual income variables had substantial missing data.

## 5 Conclusions

We found that bicycle use is increasing over time for all socio-economic groups in 18 Latin American cities between 2008 and 2018. Furthermore, individuals with high socio-economic position (high education and income level) were more likely to report lower bicycle use than individuals with lower socioeconomic position, although their use of bicycles is increasing at a faster rate than for other groups. Bicycles represent a mobility alternative with a broadening appeal that can address transportation, health, and environmental challenges. However, this wider appeal also raises concerns about the role that bicycle use and bicycle infrastructure may play in neighborhood change and population displacement. Considering policies that encourage bicycling and its infrastructure within a framework that considers equity and justice is likely to strengthen the success of bicycle- related policies.

## Supplementary Material

The Supplementary Material for this article can be found online at: https://www.frontiersin.org/articles/10.3389/frsc.2023.1055351/full#supplementary-material

Supplementary material

## Figures and Tables

**Table 1:** Study cities and participants per year.

City (by country)	2008	2009	2010	2011	2012	2013	2014	2015	2016	2017	2018
Argentina											
Buenos Aires	303	322	489	474	405	648	808	721	1,153	737	737
Cordoba	317	319	485	495	404	0	0	0	0	0	0
Bolivia											
La Paz	334	356	517	515	430	524	848	670	749	734	752
Santa Cruz	358	362	515	527	446	512	0	0	0	0	0
Brazil											
Sao Paulo	333	332	515	484	414	489	841	731	764	776	790
Rio de Janeiro	334	322	474	493	410	642	0	0	0	0	0
Colombia											
Bogota	341	332	495	502	423	669	822	730	1,135	760	779
Medellin	328	345	479	486	404	502	0	0	0	0	0
Ecuador											
Quito	334	342	515	526	429	527	860	672	673	738	750
Guayaquil	346	347	539	530	433	519	0	0	0	0	0
Mexico											
Mexico City	0	0	0	0	0	0	830	737	783	776	778
Panama											
Panama City	0	0	530	538	427	515	525	437	441	466	461
Peru											
Lima	344	343	516	510	434	532	870	693	734	747	738
Arequipa	352	346	516	507	443	0	0	0	0	0	0
Uruguay											
Montevideo	326	304	469	486	398	439	761	0	725	763	781
Salto	312	283	447	471	382	0	0	0	0	0	0
Venezuela											
Caracas	345	329	514	522	443	660	832	750	1,090	748	764
Maracaibo	333	337	502	492	433	0	0	0	0	0	0
Total	5,340	5,321	8,517	8,558	7,158	7,178	7,997	6,141	8,247	7,245	7,330

**Table 2:** Selected characteristics of the study population.

	2008 (*n* = 5,340)	2009 (*n* = 5,321)	2010 (*n* = 8,517)	2011 (*n* = 8,558)	2012 (*n* = 7,158)	2013 (*n* = 7,178)	2014 (*n* = 7,997)	2015 (*n* = 6,141)	2016 (*n* = 8,247)	2017 (*n* = 7,245)	2017 (*n* = 7,245)
	%	%	%	%	%	%	%	%	%	%	%
Age [mean (sd)]	37.89 (8.82)	38.05 (9.00)	38.21 (8.99)	38.25 (8.97)	38.13 (8.79)	38.16 (8.83)	38.30 (8.85)	38.45 (9.18)	38.48 (8.69)	38.20 (8.72)	38.49 (8.79)
Gender											
Man	48.60%	47.60%	47.80%	48.20%	48.40%	47.40%	47.00%	47.80%	46.50%	47.30%	48.60%
Woman	51.40%	52.40%	52.20%	51.80%	51.60%	52.60%	53.00%	52.20%	53.50%	52.70%	51.40%
Educational level											
Less than HS	25.70%	23.60%	42.40%	38.20%	37.00%	33.60%	34.40%	34.40%	40.00%	32.70%	30.10%
HS/ professional	54.50%	58.20%	44.50%	47.70%	49.90%	51.60%	52.60%	53.00%	47.90%	52.70%	53.20%
College or higher	19.80%	18.10%	13.10%	14.10%	13.10%	14.80%	13.10%	12.60%	12.00%	14.60%	16.60%
Missings	0.10%	0.20%	0.10%	0.20%	0%	0.20%	0.10%	0.20%	0%	2.60%	0.10%
Income level^*^											
Low	26.90%	26.90%	23.20%	14.40%	14.60%	26.00%	14.00%	19.40%	14.40%	13.00%	23.50%
Medium-low	35.80%	38.50%	44.00%	39.10%	37.20%	40.10%	34.60%	30.30%	34.10%	31.60%	32.40%
Medium-high	25.90%	25.00%	23.70%	30.50%	34.30%	22.00%	32.80%	34.10%	35.50%	37.20%	30.30%
High	11.40%	9.70%	9.10%	16.10%	13.90%	11.90%	18.60%	16.30%	15.90%	18.30%	13.80%
Missing	39.10%	50.60%	62.10%	57.80%	49.60%	45.30%	39.90%	47.90%	37.80%	46.60%	47.80%
Km bicycling infrastructure [mean (sd)]	69.81 (98.11)	75.13 (103.13)	71.32 (104.92)	77.05 (111.12)	86.33 (115.44)	106.67 (127.22)	111.61 (118.15)	135.24 (127.50)	147.90 (142.28)	145.06 (137.77)	156.08 (146.11)
Missing	48.63%	49.60%	37.96%	38.06%	43.94%	29.15%	0%	0%	0%	0%	0%
Bicycle use											
No	96.30%	98.10%	98.40%	98.50%	98.40%	98.40%	97.20%	86.60%	97.00%	94.60%	97.40%
Yes	3.70%	1.90%	1.60%	1.50%	1.60%	1.60%	2.80%	13.40%	3.00%	5.40%	2.60%
missing	1.90%	1.00%	1.00%	1.80%	1.70%	1.00%	0%	0.70%	4.60%	0%	0%

SD, standard deviation; HS, high school.

* For the surveys from 2008 to 2012 and 2014 the values were: low equivalent to ≤$200, medium-low equivalent to $201–$400, medium-high equivalent to $401–$800, and high equivalent to ≥$801. For the survey from 2013 the values were: low equivalent to ≤$100, medium-low equivalent to $101–$400, medium-high equivalent to $401–$800, and high equivalent to ≥$801. And for the surveys from 2015 to 2018 the values were: low equivalent to ≤$400, medium-low equivalent to $401–$800, medium-high equivalent to $801–$1,600, and high equivalent to ≥$1,601.

**Table 3:** Odds ratios of bike use associated with education level and individual income in single and multiple predictor models after controlling for different sets of covariates.

Socio-economic position	Model 0		Model 1	
	OR (CI 95%)	*p*-value	OR (CI 95%)	*p*-value
Education level single predictor models				
Education level		0.001		<0.001
Less than HS	Referent		Referent	
HS/professional	0.95 (0.87, 1.03)	0.209	0.88 (0.80, 0.96)	0.004
College or higher	0.78 (0.69, 0.89)	<0.001	0.70 (0.61, 0.80)	<0.001
Age			0.99 (0.98, 0.99)	<0.001
Gender (woman)			0.38 (0.35, 0.41)	<0.001
Income level single predictor models				
Income level^*^		0.009		<0.001
Low	Referent		Referent	
Medium-low	1.22 (1.04, 1.43)	0.014	0.98 (0.83, 1.15)	0.773
Medium-high	1.14(0.97, 1.35)	0.104	0.80 (0.68, 0.95)	0.011
High	0.96 (0.79, 1.16)	0.680	0.65 (0.53, 0.79)	<0.001
Age			0.99 (0.98, 0.99)	<0.001
Gender (woman)			0.37 (0.32, 0.41)	<0.001

HS, high school.

* For the surveys from 2008 to 2012 and 2014 the values were: low equivalent to ≤$200, medium-low equivalent to $201–$400, medium-high equivalent to $401–$800, and high equivalent to ≥$801. For the survey from 2013 the values were: low equivalent to ≤$100, medium-low equivalent to $101–$400, medium-high equivalent to $401–$800, and high equivalent to ≥$801. And for the surveys from 2015 to 2018 the values were: low equivalent to ≤$400, medium-low equivalent to $401–$800, medium-high equivalent to $801–$1,600, and high equivalent to ≥$1,601.

Model 0 definition: model without covariates; city as random intercept and year as random slope. Model 1 definition: model after controlling for age and gender; city as random intercept and year as random slope. Sample size education level single predictor models: Model 0 (n = 77,765); Model 1 (n = 77,765). Sample size income level single predictor models: Model 0 (n = 40,545); Model 1 (n = 40,545).

## Data Availability

The SALURBAL project welcomes queries from anyone interested in learning more about its dataset and potential access to data. To learn more about SALURBAL’s dataset, visit the SALURBAL project website or contact the project at salurbal@drexel.edu. After publication of this study, study protocols, data dictionaries, and requested study data may be made available to interested investigators after they have signed a data use agreement with SALURBAL and if their study proposal, developed in collaboration with SALURBAL investigators, is approved by the SALURBAL proposal and publications committee. Some data may not be available to external investigators because of data confidentiality agreements.
