# Supplementary material for: Bicycle use in Latin American cities: changes over time by socio-economic position
Source: Front Sustain Cities. Author manuscript; Available in PMC 2024 Dec 5. (PMC7616888; doi:10.3389/frsc.2023.1055351)
Supplement: Supplementary material [file EMS177352-supplement-Supplementary_material.docx]

Supplementary Material

# Figure A. Directed Acyclic Graph.


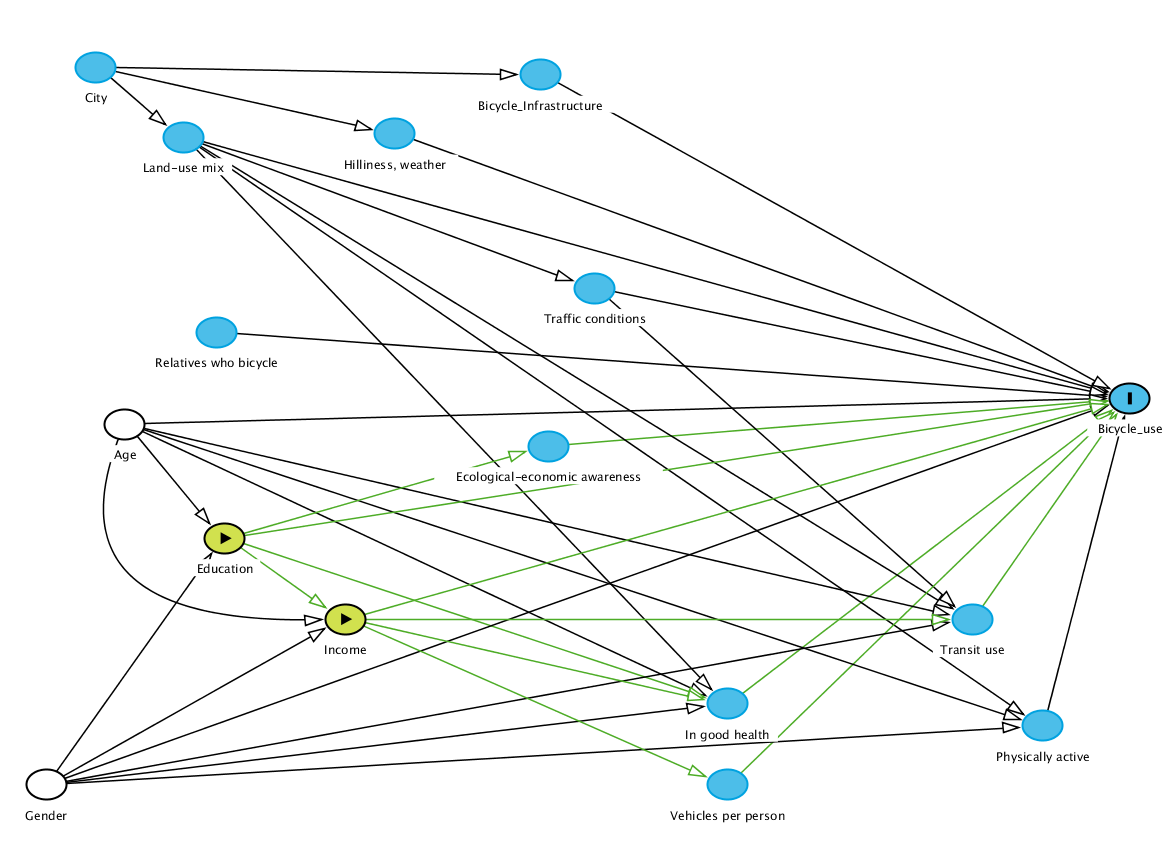


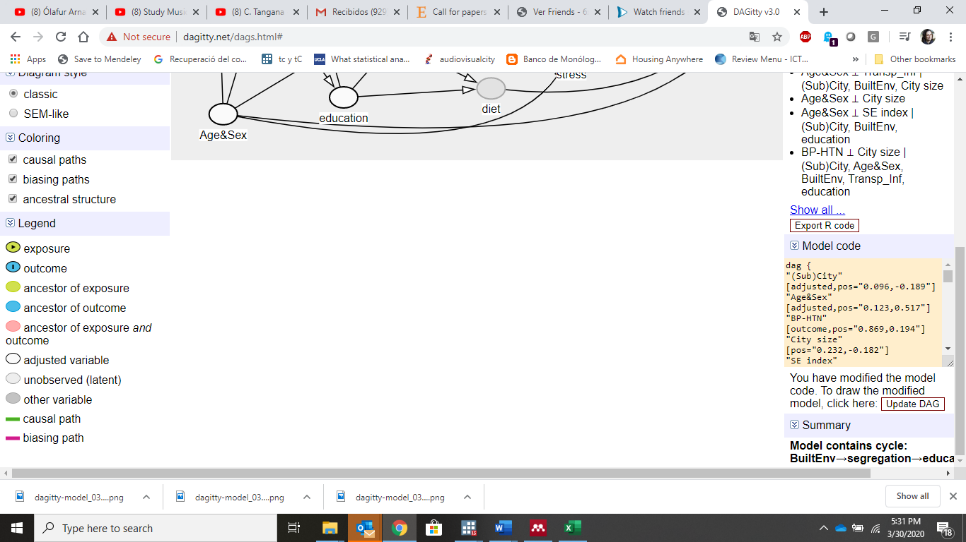

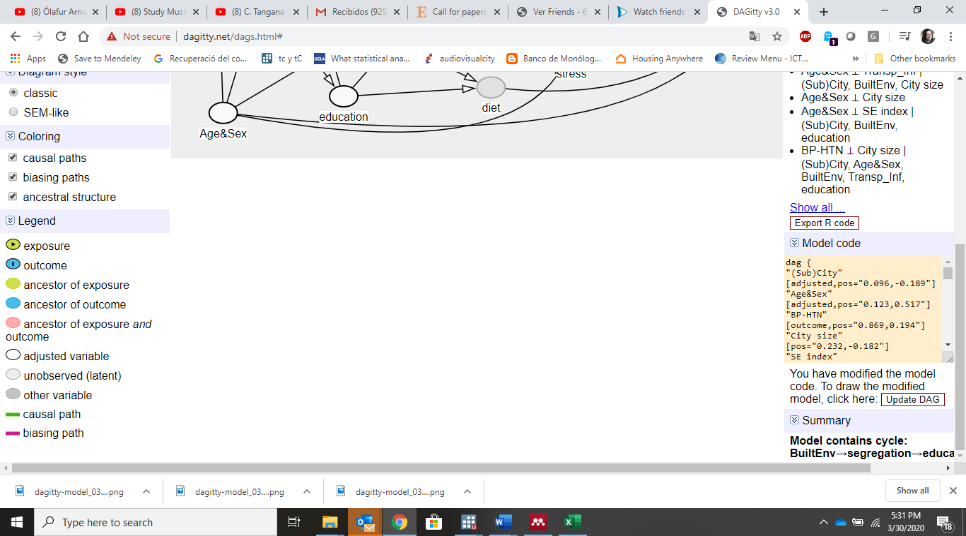

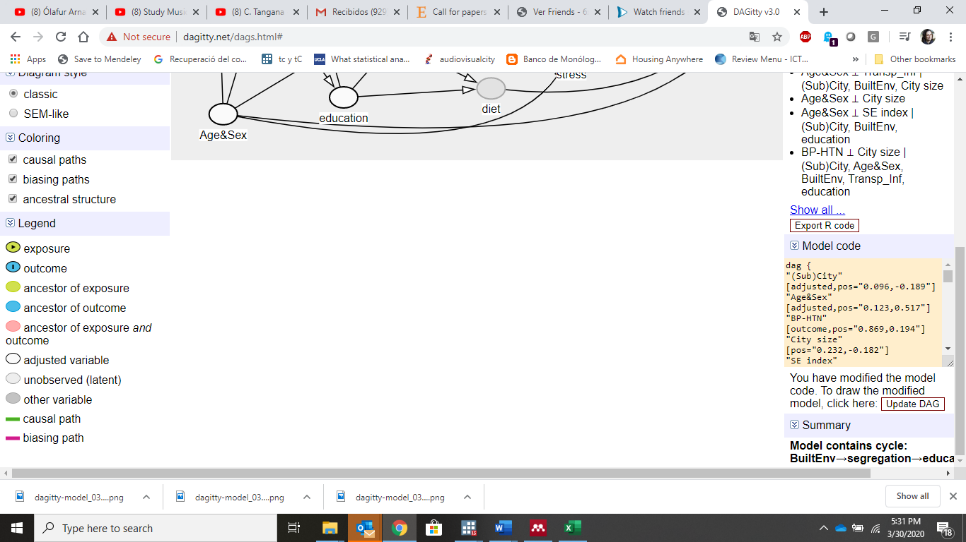


The minimal sufficient adjustment set for estimating the total effect of the exposures on the outcomes were: age and gender.

**Table A. Length of the infrastructure dedicated to bicycle per city.**

| **City (Country)** | **km** | **Year 1** | **Source** | **km** | **Year 2** | **Source** |
| --- | --- | --- | --- | --- | --- | --- |
| Buenos Aires (Argentina) | 93 | 2007 | (Observatorio Urbano de Movilidad. Development Bank of Latin America, Caracas, Venezuela., 2007) | 202 | 2018 | (Pardo, Carlosfelipe; Rodriguez, Daniel A.; Quiñones, 2021) |
| Bogota (Colombia) | 291 | 2007 | (Observatorio Urbano de Movilidad. Development Bank of Latin America, Caracas, Venezuela., 2007) | 500 | 2018 | (Pardo, Carlosfelipe; Rodriguez, Daniel A.; Quiñones, 2021) |
| Caracas (Venezuela) | 14 | 2007 | (Observatorio Urbano de Movilidad. Development Bank of Latin America, Caracas, Venezuela., 2007) | 26 | 2018 | (Pardo, Carlosfelipe; Rodriguez, Daniel A.; Quiñones, 2021) |
| La Paz (Bolivia) | 2 | 2013 | (Bank, 2015) | 14 | 2018 | (Pardo, Carlosfelipe; Rodriguez, Daniel A.; Quiñones, 2021) |
| Lima (Peru) | 59 | 2007 | (Observatorio Urbano de Movilidad. Development Bank of Latin America, Caracas, Venezuela., 2007) | 180 | 2018 | (Pardo, Carlosfelipe; Rodriguez, Daniel A.; Quiñones, 2021) |
| Mexico City (Mexico) | 30 | 2007 | (Observatorio Urbano de Movilidad. Development Bank of Latin America, Caracas, Venezuela., 2007) | 170 | 2018 | (Pardo, Carlosfelipe; Rodriguez, Daniel A.; Quiñones, 2021) |
| Montevideo (Uruguay) | 8 | 2007 | (Observatorio Urbano de Movilidad. Development Bank of Latin America, Caracas, Venezuela., 2007) | 35 | 2018 | (Pardo, Carlosfelipe; Rodriguez, Daniel A.; Quiñones, 2021) |
| Panamá City (Panama) | 15.8 | 2013 | (Bank, 2015) | 35 | 2018 | (Pardo, Carlosfelipe; Rodriguez, Daniel A.; Quiñones, 2021) |
| Quito (Ecuador) | 46 | 2007 | (Observatorio Urbano de Movilidad. Development Bank of Latin America, Caracas, Venezuela., 2007) | 70.54 | 2018 | (Pardo, Carlosfelipe; Rodriguez, Daniel A.; Quiñones, 2021) |
| Sao Paulo (Brazil) | 68.3 | 2013 | (Baumman, C. Bojaca, M., Rambeaus, M. and Wanner, 2013) | 270.71 | 2018 | (Pardo, Carlosfelipe; Rodriguez, Daniel A.; Quiñones, 2021) |

Bank, I. D. (2015). Fichas de acciones de mitigacion, Inicitativa de Ciudades Emergentes y Sostenibles. Available at: https://dpu.mupa.gob.pa/wp-content/uploads/2017/06/Anexo-1-Fichas-acciones-de-mitigacion_Panama.pdf [Accessed September 18, 2019].

Baumman, C. Bojaca, M., Rambeaus, M. and Wanner, Z. (2013). Biciudades 2013: un estudio regional acerca del uso de la bicicleta como medio de transporte en América Latina y el Caribe, Washington DC.

Observatorio Urbano de Movilidad. Development Bank of Latin America, Caracas, Venezuela. (2007).

Pardo, Carlosfelipe; Rodriguez, Daniel A.; Quiñones, L. M. (2021). “Cycling in Latin America,” in *Cycling for Sustainable Cities*, ed. J. Buehler, R. and Pucher (MIT Press, Cambridge, MA.).

**Table B. Equation of the multilevel logistic regression model used to evaluate how education level is associated to bicycle use in adults in Latin American cities.**

| **First level (individual) equation:** |
| --- |
| Log (odds bike_ij_) = *b*_0j_ + *b*_1_ education HS/professional _ij_ + *b_2_* education College or higher _ij_ + *b*_3_ age _ij_ + *b*_4_ gender _ij_ + *b*_5j_ year _ij_ |
| **Second level (city) equation:** |
| *b*_0j_  = γ_0_ + *U*_0j_ |
| *b*_1j_  = γ_1_ + *U*_1j_ |
| Where: |
| *U*_0j_ ~ *N* (0, σ_0_^2^) |
| *U*_1j_ ~ *N* (0, σ_1_^2^) |
| Cov (*U*_0j_, *U*_1j_) = 0 |

i^th^ individual in j^th^ city.

γ_0_= Global intercept

*U*_0j_=City specific intercept

*b*_1j_=Global specific slope

γ_1_=Global trend

*U*_1j_=City specific trend

**Table C. Distribution of bicycle use according to city and year of survey**

| **% Bicycle use** | **2008** | **2009** | **2010** | **2011** | **2012** | **2013** | **2014** | **2015** | **2016** | **2017** | **2018** |
| --- | --- | --- | --- | --- | --- | --- | --- | --- | --- | --- | --- |
| **Argentina** |  |  |  |  |  |  |  |  |  |  |  |
| Buenos Aires | 5.96% | 1.56% | 6.47% | 3.23% | 5.99% | 4.34% | 5.82% | 9.21% | 4.65% | 8.14% | 3.80% |
| Cordoba | 4.11% | 3.46% | 2.68% | 3.23% | 1.49% |  |  |  |  |  |  |
| **Bolivia** |  |  |  |  |  |  |  |  |  |  |  |
| La Paz | 0.62% | 0% | 0% | 0% | 0.24% | 0% | 0.24% | 17.84% | 0.55% | 1.91% | 0.53% |
| Santa Cruz | 1.12% | 1.10% | 0.59% | 0.59% | 1.81% | 1.19% |  |  |  |  |  |
| **Brazil** |  |  |  |  |  |  |  |  |  |  |  |
| Sao Paulo | 0.60% | 0.91% | 0.59% | 1.45% | 0.97% | 0.41% | 2.26% | 8.21% | 1.47% | 3.61% | 1.14% |
| Rio de Janeiro | 8.68% | 2.17% | 1.27% | 2.03% | 1.71% | 2.50% |  |  |  |  |  |
| **Colombia** |  |  |  |  |  |  |  |  |  |  |  |
| Bogota | 10.90% | 3.85% | 3.85% | 4.41% | 5.46% | 4.12% | 3.89% | 24.45% | 7.68% | 16.18% | 9.24% |
| Medellin | 1.23% | 2.03% | 1.11% | 0.21% | 0.25% | 0.60% |  |  |  |  |  |
| **Ecuador** |  |  |  |  |  |  |  |  |  |  |  |
| Quito | 0.60% | 0.29% | 0.97% | 0.57% | 0.94% | 0.19% | 1.63% | 13.69% | 0.63% | 3.52% | 1.33% |
| Guayaquil | 0.88% | 1.73% | 0.56% | 0% | 0.69% | 2.57% |  |  |  |  |  |
| **Mexico** |  |  |  |  |  |  |  |  |  |  |  |
| Mexico City |  |  |  |  |  |  | 6.51% | 24.69% | 3.59% | 6.19% | 1.54% |
| **Panama** |  |  |  |  |  |  |  |  |  |  |  |
| Panama City |  |  | 0.19% | 0% | 0.48% | 0% | 0.19% | 5.26% | 0% | 0.64% | 0.22% |
| **Peru** |  |  |  |  |  |  |  |  |  |  |  |
| Lima | 1.83% | 0.88% | 0.39% | 1.79% | 0.23% | 0.38% | 0.80% | 9.71% | 1.70% | 2.68% | 0.81% |
| Arequipa | 0.85% | 0.58% | 0.19% | 1.59% | 0.45% |  |  |  |  |  |  |
| **Uruguay** |  |  |  |  |  |  |  |  |  |  |  |
| Montevideo | 11.15% | 3.99% | 3.91% | 3.79% | 3.54% | 3.50% | 5.65% | 5.77% | 8.26% | 5.76% |  |
| Salto | 12.70% | 7.53% | 2.24% | 3.65% | 3.17% |  |  |  |  |  |  |
| **Venezuela** |  |  |  |  |  |  |  |  |  |  |  |
| Caracas | 0% | 0% | 0.39% | 0.19% | 0% | 0.31% | 0.84% | 4.14% | 0.19% | 1.20% | 0.26% |
| Maracaibo | 0% | 1.79% | 1.81% | 0.61% | 1.41% |  |  |  |  |  |  |

**Table D.** **Odds ratios of bike use associated with education level and individual income in multiple predictor models after controlling for different sets of covariates.**

|  | **Model 0** |  | **Model 1** |  |
| --- | --- | --- | --- | --- |
| **Socio-economic position** | **OR (CI 95%)** | **p-value** | **OR (CI 95%)** | **p-value** |
| Multiple predictors models |  |  |  |  |
| Education level |  | 0.112 |  | 0.512 |
| Less than HS | referent |  | referent |  |
| HS/professional | 0.96 (0.85, 1.08) | 0.453 | 0.95 (0.84, 1.08) | 0.445 |
| College or higher | 0.83 (0.69, 0.99) | 0.037 | 0.90 (0.75, 1.08) | 0.261 |
| Income level* |  | 0.031 |  | <0.001 |
| Low | referent |  | referent |  |
| Medium-low | 1.23 (1.05, 1.44) | 0.011 | 0.98 (0.83, 1.15) | 0.814 |
| Medium-high | 1.18 (0.99, 1.39) | 0.053 | 0.82 (0.69, 0.97) | 0.020 |
| High | 1.03 (0.84, 1.26) | 0.775 | 0.67 (0.54, 0.83) | <0.001 |
| Age |  |  | 0.99 (0.98, 0.99) | <0.001 |
| Gender (Woman) |  |  | 0.37 (0.32, 0.42) | <0.001 |

HS, High School

* For the surveys from 2008 to 2012 and 2014 the values were: low equivalent to <=$200, medium-low equivalent to $201-$400, medium-high equivalent to $401-$800, and high equivalent to >=$801. For the survey from 2013 the values were: low equivalent to <=$100, medium-low equivalent to $101-$400, medium-high equivalent to $401-$800, and high equivalent to >=$801. And for the surveys from 2015 to 2018 the values were: low equivalent to <=$400, medium-low equivalent to $401-$800, medium-high equivalent to $801-$1600, and high equivalent to >=$1601.

Model 0 definition: model without covariates; city as random intercept and year as random slope.

Model 1 definition: model after controlling for age and gender; city as random intercept and year as random slope.

Sample sizes: Model 0 (n=40,545); Model 1 (n=40,545).

**Figure B. Predictive marginal probabilities of bike use by education and income level over time in CAF surveys of select Latin America cities. Multiple predictors models after controlling for age and gender (model 1).**

A) B)

HS, High School

1. Education level multiple predictors model after controlling for age and gender (model 1). Global test interaction p-value=0.004. 95% CIs are not displayed for clarity.
2. Income level multiple predictors model after controlling for age and gender (model 1). Global test interaction p-value<0.001. 95% CIs are not displayed for clarity.

**Table E.** **Odds ratios of bike use associated with education level and individual income in single and multiple exposure models after controlling for age, gender, km of bicycling infrastructure.**

|  | **Model 2** |  |
| --- | --- | --- |
| **Socio-economic position** | **OR (CI 95%)** | **p-value** |
| Education level single predictor models | |  |
| Education level |  | 0.001 |
| Less than HS | referent |  |
| HS/professional | 0.96 (0.87, 1.05) | 0.359 |
| College or higher | 0.77 (0.67, 0.89) | <0.001 |
| Age | 0.98 (0.98, 0.99) | <0.001 |
| Gender (Woman) | 0.41 (0.37, 0.45) | <0.001 |
| Km bicycling infrastructure | 1.00 (0.99, 1.00) | 0.218 |
| Income level single predictor models | |  |
| Income level* |  | 0.002 |
| Low | referent |  |
| Medium-low | 1.05 (0.88, 1.25) | 0.590 |
| Medium-high | 0.90 (0.75, 1.08) | 0.255 |
| High | 0.75 (0.61, 0.93) | 0.008 |
| Age | 0.98 (0.98, 0.99) | <0.001 |
| Gender (Woman) | 0.39 (0.34, 0.44) | <0.001 |
| Km bicycling infrastructure | 0.99 (0.99, 1.00) | 0.112 |
| Multiple predictors models |  |  |
| Education level |  | 0.632 |
| Less than HS | referent |  |
| HS/professional | 1.06 (0.93, 1.21) | 0.410 |
| College or higher | 1.00 (0.82, 1.21) | 0.979 |
| Income level* |  | 0.004 |
| Low | referent |  |
| Medium-low | 1.05 (0.88, 1.25) | 0.616 |
| Medium-high | 0.89 (0.74, 1.08) | 0.242 |
| High | 0.75 (0.60, 0.94) | 0.013 |
| Age | 0.98 (0.98, 0.99) | <0.001 |
| Gender (Woman) | 0.39 (0.34, 0.44) | <0.001 |
| Km bicycling infrastructure | 0.99 (0.99, 1.00) | 0.111 |

HS, High School

* For the surveys from 2008 to 2012 and 2014 the values were: low equivalent to <=$200, medium-low equivalent to $201-$400, medium-high equivalent to $401-$800, and high equivalent to >=$801. For the survey from 2013 the values were: low equivalent to <=$100, medium-low equivalent to $101-$400, medium-high equivalent to $401-$800, and high equivalent to >=$801. And for the surveys from 2015 to 2018 the values were: low equivalent to <=$400, medium-low equivalent to $401-$800, medium-high equivalent to $801-$1600, and high equivalent to >=$1601.

Model 2 definition: model after controlling for age, gender, and Km of separated bicycling infrastructure; city as random intercept and year as random slope.

Sample size education level single predictor models: Model 2 (n=59,113). Sample size income level single predictor models & multiple predictors models: Model 2 (n=32,382)

**Figure C. Predictive marginal probabilities of bike use by education and income level over time in CAF surveys of select Latin America cities. Models after controlling for age, gender, and kilometers of bicycling infrastructure (model 2).**

A) B)

C) D)

HS, High School

1. Education level single predictor model after controlling for age, gender, and kilometers of bicycling infrastructure (model 2). Global test interaction p-value<0.001. 95% CIs are not displayed for clarity.
2. Income level single predictor model after controlling for age, gender, and kilometers of bicycling infrastructure (model 2). Global test interaction p-value<0.001. 95% CIs are not displayed for clarity.
3. Education level multiple predictors model after controlling for age, gender, and kilometers of bicycling infrastructure (model 2). Global test interaction p-value=0.0809. 95% CIs are not displayed for clarity.
4. Income level multiple predictors model after controlling for age, gender, and kilometers of bicycling infrastructure (model 2). Global test interaction p-value<0.001. 95% CIs are not displayed for clarity.

**Table F.** **Odds ratios of bike use associated with education level and individual income in single and multiple predictor models after controlling for different sets of covariates and excluding 2015 sample.**

|  | **Model 0** |  | **Model 1** |  |
| --- | --- | --- | --- | --- |
| **Socio-economic status** | **OR (CI 95%)** | **p-value** | **OR (CI 95%)** | **p-value** |
| Education level single predictor models | |  |  |  |
| Education level |  | <0.001 |  | <0.001 |
| Less than HS | referent |  | referent |  |
| HS/professional | 0.91 (0.82, 1.01) | 0.083 | 0.86 (0.77, 0.95) | 0.004 |
| College or higher | 0.70 (0.60, 0.83) | <0.001 | 0.63 (0.54, 0.75) | <0.001 |
| Age |  |  | 0.99 (0.98, 0.99) | <0.001 |
| Gender (Woman) |  |  | 0.35 (0.31, 0.38) | <0.001 |
| Income level single predictor models | |  |  |  |
| Income level* |  | <0.001 |  | <0.001 |
| Low | referent |  | Referent |  |
| Medium-low | 1.34 (1.11, 1.62) | 0.002 | 1.07 (0.88, 1.29) | 0.513 |
| Medium-high | 1.09 (0.90, 1.33) | 0.368 | 0.75 (0.61, 0.92) | 0.006 |
| High | 0.84 (0.67, 1.06) | 0.151 | 0.55 (0.44, 0.70) | <0.001 |
| Age |  |  | 0.99 (0.98, 1.00) | 0.001 |
| Gender (Woman) |  |  | 0.35 (0.30, 0.41) | <0.001 |
| Multiple predictors models |  |  |  |  |
| Education level |  | <0.001 |  | <0.001 |
| Less than HS | referent |  | referent |  |
| HS/professional | 0.96 (0.84, 1.10) | 0.574 | 0.97 (0.84, 1.12) | 0.699 |
| College or higher | 0.85 (0.68, 1.05) | 0.127 | 0.94 (0.76, 1.17) | 0.566 |
| Income level* |  |  |  |  |
| Low | referent |  | referent |  |
| Medium-low | 1.35 (1.12, 1.63) | 0.002 | 1.07 (0.88, 1.30) | 0.500 |
| Medium-high | 1.12 (0.92, 1.37) | 0.267 | 0.76 (0.62, 0.93) | 0.009 |
| High | 0.90 (0.70, 1.14) | 0.381 | 0.57 (0.44, 0.73) | <0.001 |
| Age |  |  | 0.99 (0.98, 1.00) | 0.001 |
| Gender (Woman) |  |  | 0.35 (0.30, 0.41) | <0.001 |

HS, High School

* For the surveys from 2008 to 2012 and 2014 the values were: low equivalent to <=$200, medium-low equivalent to $201-$400, medium-high equivalent to $401-$800, and high equivalent to >=$801. For the survey from 2013 the values were: low equivalent to <=$100, medium-low equivalent to $101-$400, medium-high equivalent to $401-$800, and high equivalent to >=$801. And for the surveys from 2016 to 2018 the values were: low equivalent to <=$400, medium-low equivalent to $401-$800, medium-high equivalent to $801-$1600, and high equivalent to >=$1601.

Model 0 definition: model without covariates; city as random intercept and year as random slope.

Model 1 definition: model after controlling for age and gender; city as random intercept and year as random slope.

Sample sizes: Model 0 (n=71,682); Model 1 (n=37,373).

**Table G.** **Odds ratios of bike use associated with education level in single exposure models after controlling for different sets of covariates excluding income missing data.**

|  | **Model 0** |  | **Model 1** |  | **Model 2** |  |
| --- | --- | --- | --- | --- | --- | --- |
| **Socio-economic position** | **OR (CI 95%)** | **p-value** | **OR (CI 95%)** | **p-value** | **OR (CI 95%)** | **p-value** |
| Education level single predictor models | |  |  |  |  |  |
| Education level |  | 0.033 |  | 0.011 |  | 0.191 |
| Less than HS | referent |  | referent |  | referent |  |
| HS/professional | 0.96 (0.85, 1.08) | 0.480 | 0.91 (0.81, 1.03) | 0.133 | 1.02 (0.90, 1.16) | 0.752 |
| College or higher | 0.80 (0.68, 0.95) | 0.010 | 0.77 (0.65, 0.92) | 0.003 | 0.88 (0.73, 1.05) | 0.153 |
| Age |  |  | 0.99 (0.98, 0.99) | <0.001 | 0.98 (0.98, 0.99) | <0.001 |
| Gender (Woman) |  |  | 0.39 (0.35, 0.44) | <0.001 | 0.41 (0.36, 0.46) | <0.001 |
| Km bicycling infrastructure |  |  |  |  | 1.00 (0.99, 1.00) | 0.150 |

HS, High School

Model 0 definition: model without covariates; city as random intercept and year as random slope.

Model 1 definition: model after controlling for age and gender; city as random intercept and year as random slope.

Model 2 definition: model after controlling for age, gender, and Km of separated bicycling infrastructure; city as random intercept and year as random slope.

Sample sizes: Model 0 (n=40,545); Model 1 (n=40,545); Model 2 (n=32,382).

**Figure D. Predictive marginal probabilities of bike use by education level over time in CAF surveys of select Latin America cities. Models after controlling for age and gender (model 1) and for age, gender, and kilometers of bicycling infrastructure (model 2). Sample excluding income missing data.**

A) B)

1. Education level single predictor model after controlling for age and gender (model 1). Global test interaction p-value<0.001. 95% CIs are not displayed for clarity.
2. Education level single predictor model after controlling for age, gender, and kilometers of bicycling infrastructure (model 2). Global test interaction p-value=0.025. 95% CIs are not displayed for clarity.

**Table H.** **Odds ratios of bike use associated with education level and individual income in single and multiple predictor models after controlling for different sets of covariates. Sample excluding km of bicycling infrastructure missing data.**

|  | **Model 0** |  | **Model 1** |  |
| --- | --- | --- | --- | --- |
| **Socio-economic position** | **OR (CI 95%)** | **p-value** | **OR (CI 95%)** | **p-value** |
| Education level single predictor models | |  |  |  |
| Education level |  | 0.031 |  | 0.001 |
| Less than HS | referent |  | referent |  |
| HS/professional | 1.04 (0.94, 1.14) | 0.468 | 0.96 (0.87, 1.05) | 0.350 |
| College or higher | 0.87 (0.75, 0.99) | 0.045 | 0.77 (0.67, 0.89) | <0.001 |
| Age |  |  | 0.98 (0.98, 0.99) | <0.001 |
| Gender (Woman) |  |  | 0.41 (0.37, 0.45) | <0.001 |
| Income level single predictor models | |  |  |  |
| Income level* |  | 0.014 |  | 0.002 |
| Low | referent |  | referent |  |
| Medium-low | 1.28 (1.07, 1.52) | 0.006 | 1.05 (0.88, 1.25) | 0.617 |
| Medium-high | 1.25 (1.04, 1.49) | 0.016 | 0.90 (0.75, 1.08) | 0.253 |
| High | 1.07 (0.87, 1.32) | 0.505 | 0.75 (0.61, 0.93) | 0.008 |
| Age |  |  | 0.98 (0.98, 0.99) | <0.001 |
| Gender (Woman) |  |  | 0.39 (0.34, 0.44) | <0.001 |
| Multiple predictors models |  |  |  |  |
| Education level |  | 0.218 |  | 0.637 |
| Less than HS | referent |  | referent |  |
| HS/professional | 1.06 (0.94, 1.21) | 0.345 | 1.05 (0.92, 1.20) | 0.435 |
| College or higher | 0.92 (0.76, 1.12) | 0.404 | 0.99 (0.82, 1.20) | 0.926 |
| Income level* |  | 0.025 |  | 0.006 |
| Low | referent |  | referent |  |
| Medium-low | 1.28 (1.07, 1.52) | 0.006 | 1.04 (0.87, 1.25) | 0.642 |
| Medium-high | 1.25 (1.04, 1.50) | 0.015 | 0.89 (0.74, 1.08) | 0.245 |
| High | 1.11 (0.89, 1.38) | 0.353 | 0.75 (0.60, 0.94) | 0.014 |
| Age |  |  | 0.98 (0.98, 0.99) | <0.001 |
| Gender (Woman) |  |  | 0.39 (0.34, 0.44) | <0.001 |

HS, High School

* For the surveys from 2008 to 2012 and 2014 the values were: low equivalent to <=$200, medium-low equivalent to $201-$400, medium-high equivalent to $401-$800, and high equivalent to >=$801. For the survey from 2013 the values were: low equivalent to <=$100, medium-low equivalent to $101-$400, medium-high equivalent to $401-$800, and high equivalent to >=$801. And for the surveys from 2015 to 2018 the values were: low equivalent to <=$400, medium-low equivalent to $401-$800, medium-high equivalent to $801-$1600, and high equivalent to >=$1601.

Model 0 definition: model without covariates; city as random intercept and year as random slope. Model 1 definition: model after controlling for age and gender; city as random intercept and year as random slope.

Sample size education level single predictor models: Model 0 (n=59,113); Model 1 (n=59,113). Sample size income level single predictor models & multiple predictors models: Model 0 (n=32,382); Model 1 (n=32,382).

**Figure E. Predictive marginal probabilities of bike use by education and income level over time in CAF surveys of select Latin America cities. Models after controlling for age and gender (model 1). Sample excluding km of bicycling infrastructure missing data.**

A) B)

C) D)

HS, High School

1. Education level single predictor model after controlling for age and gender (model 1). Global test interaction p-value<0.001. 95% CIs are not displayed for clarity.
2. Income level single predictor model after controlling for age and gender (model 1). Global test interaction p-value<0.001. 95% CIs are not displayed for clarity.
3. Multiple predictors model after controlling for age and gender (model 1). Global test interaction p-value= 0.1912. 95% CIs are not displayed for clarity.
4. Multiple predictors model after controlling for age and gender (model 1). Global test interaction p-value<0.001. 95% CIs are not displayed for clarity.

**Table I.** **Odds ratios of bike use associated with education level in single predictor models after controlling for different sets of covariates. Sample excluding income and km of bicycling infrastructure missing data.**

|  | **Model 0** |  | **Model 1** |  |
| --- | --- | --- | --- | --- |
| **Socio-economic position** | **OR (CI 95%)** | **p-value** | **OR (CI 95%)** | **p-value** |
| Education level single predictor models | |  |  |  |
| Education level |  | 0.117 |  | 0.184 |
| Less than HS | referent |  | referent |  |
| HS/professional | 1.08 (0.95, 1.22) | 0.259 | 1.02 (0.89, 1.16) | 0.776 |
| College or higher | 0.91 (0.76, 1.09) | 0.310 | 0.87 (0.73, 1.05) | 0.142 |
| Age |  |  | 0.98 (0.98, 0.99) | <0.001 |
| Gender (Woman) |  |  | 0.41 (0.36, 0.46) | <0.001 |

HS, High School

Model 0 definition: model without covariates; city as random intercept and year as random slope.

Model 1 definition: model after controlling for age and gender; city as random intercept and year as random slope.

Sample sizes: Model 0 (n= 32,382); Model 1 (n=32,382).

**Figure F. Predictive marginal probabilities of bike use by education level over time in CAF surveys of select Latin America cities. Models after controlling for age and gender (model 1). Sample excluding income and km of bicycling infrastructure missing data.**

Education level single predictor model after controlling for age and gender (model 1). Global test interaction p-value=0.0506. 95% CIs are not displayed for clarity.

**Table J. Distribution between education and income level.**

|  | **Total (n=79,032)** | **Educational level** | | | | |
| --- | --- | --- | --- | --- | --- | --- |
|  |  | **Less than HS (n= 27,227)** | **HS/professional (n=40,147)** | **College or higher (n=11,393)** | **Missing (n=265)** | **p-value*** |
|  | **%** | **%** | **%** | **%** | **%** |  |
| Income level |  |  |  |  |  | <0.001 |
| Low | 9.9 | 12.3 | 9.6 | 5.3 | 5.7 |  |
| Medium-low | 18.7 | 19.3 | 19.7 | 13.5 | 15.5 |  |
| Medium-high | 15.9 | 12.1 | 17.3 | 19.9 | 20 |  |
| High | 7.5 | 3.7 | 6.4 | 20.7 | 10.6 |  |
| missing | 48 | 52.5 | 47 | 40.6 | 48.3 |  |

*Chi-square test

HS, High School

**Table K. Distribution between education and income level with complete case analyses.**

|  | **Total (n=40,949)** | **Educational level** | | | |
| --- | --- | --- | --- | --- | --- |
|  |  | **Less than HS (n= 12,926)** | **HS/professional (n=21,260)** | **College or higher (n=6,763)** | **p-value*** |
|  | **%** | **%** | **%** | **%** |  |
| Income level |  |  |  |  | <0.001 |
| Low | 19.1 | 26 | 18.1 | 9 |  |
| Medium-low | 36 | 40.7 | 37.3 | 22.7 |  |
| Medium-high | 30.5 | 25.6 | 32.6 | 33.4 |  |
| High | 14.4 | 7.7 | 12 | 34.9 |  |

*Chi test

HS, High School
